# 5-Diethyl­amino-2-[(*E*)-(4-eth­oxy­phen­yl)imino­meth­yl]phenol

**DOI:** 10.1107/S1600536811004533

**Published:** 2011-02-12

**Authors:** Erkan Soydemir, Orhan Büyükgüngör, Çiğdem Albayrak, Mustafa Odabaşoğlu

**Affiliations:** a Department of Physics, Ondokuz Mayıs University, TR-55139 Samsun, Turkey; b Sinop Faculty of Education, Sinop University, Sinop, Turkey; c Chemistry Programme, Denizli Higher Vocational School, Pamukkale University, TR-20159 Denizli, Turkey

## Abstract

The title compound, C_19_H_24_N_2_O_2_, adopts the phenol–imine tautomeric form. An intra­molecular O—H⋯N hydrogen bond results in the formation of a six-membered ring. The aromatic rings are oriented at a dihedral angle of 17.33 (16)°. Inter­molecular C—H⋯π inter­actions occur in the crystal.

## Related literature

For general background to Schiff bases, see: Hadjoudis *et al.* (1987 ▶); Hodnett & Dunn (1970 ▶); Misra *et al.* (1981 ▶); Agarwal *et al.* (1983 ▶); Varma *et al.* (1986 ▶); Singh & Dash (1988 ▶); Pandeya *et al.* (1999 ▶); El-Masry *et al.* (2000 ▶); Cohen *et al.* (1964 ▶); Moustakali-Mavridis *et al.* (1978 ▶) Kaitner & Pavlovic (1996 ▶); Yıldız *et al.* (1998 ▶). For related structures, see: Odabaşoğlu *et al.* (2003 ▶); Hökelek *et al.* (2000 ▶); Bingöl Alpaslan *et al.* (2010 ▶).
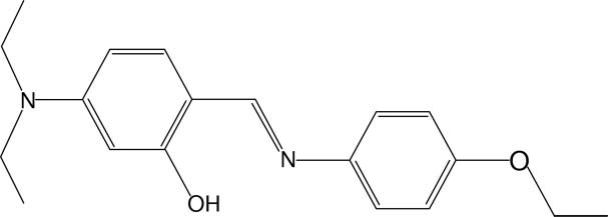


## Experimental

### 

#### Crystal data

C_19_H_24_N_2_O_2_
*M* *_r_* = 312.40
Monoclinic, 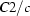

*a* = 29.4936 (13) Å
*b* = 7.8546 (2) Å
*c* = 16.7146 (7) Å
β = 115.093 (3)°
*V* = 3506.7 (2) Å^3^
*Z* = 8
Mo *K*α radiation
μ = 0.08 mm^−1^
*T* = 296 K
0.76 × 0.59 × 0.28 mm

#### Data collection

Stoe IPDS 2 diffractometer
Absorption correction: integration (*X-RED32*; Stoe & Cie, 2002 ▶) *T*_min_ = 0.944, *T*_max_ = 0.979
22701 measured reflections
3625 independent reflections
2383 reflections with *I* > 2σ(*I*)
*R*_int_ = 0.073

#### Refinement

*R*[*F* ^2^ > 2σ(*F* ^2^)] = 0.080
*wR*(*F* ^2^) = 0.260
*S* = 1.10
3625 reflections
208 parameters
4 restraints
H-atom parameters constrained
Δρ_max_ = 0.56 e Å^−3^
Δρ_min_ = −0.28 e Å^−3^

### 

Data collection: *X-AREA* (Stoe & Cie, 2002 ▶); cell refinement: *X-AREA*; data reduction: *X-RED* (Stoe & Cie, 2002 ▶); program(s) used to solve structure: *SHELXS97* (Sheldrick, 2008 ▶); program(s) used to refine structure: *SHELXL97* (Sheldrick, 2008 ▶); molecular graphics: *ORTEP-3 for Windows* (Farrugia, 1997 ▶); software used to prepare material for publication: *WinGX* (Farrugia, 1999 ▶).

## Supplementary Material

Crystal structure: contains datablocks I, global. DOI: 10.1107/S1600536811004533/fj2390sup1.cif

Structure factors: contains datablocks I. DOI: 10.1107/S1600536811004533/fj2390Isup2.hkl

Additional supplementary materials:  crystallographic information; 3D view; checkCIF report

## Figures and Tables

**Table 1:** Hydrogen-bond geometry (Å, °) *Cg*1 is the centroid of C8–C13 ring.

*D*—H⋯*A*	*D*—H	H⋯*A*	*D*⋯*A*	*D*—H⋯*A*
O1—H1⋯N1	0.82	1.88	2.610 (3)	148
C2—H2⋯*Cg*1^i^	0.93	2.85	3.681 (4)	149
C17—H17*A*⋯*Cg*1^ii^	0.96	2.97	3.763 (6)	140

Symmetry codes: (i) 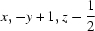; (ii) 
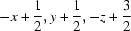
.

**Fig. 1.**
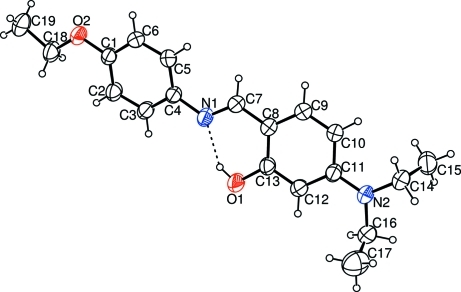
An ORTEP view of (I), with the atom-numbering scheme and 30% probability displacement ellipsoids. The dashed line indicates the intramolecular hydrogen bond.

**Fig. 2.**
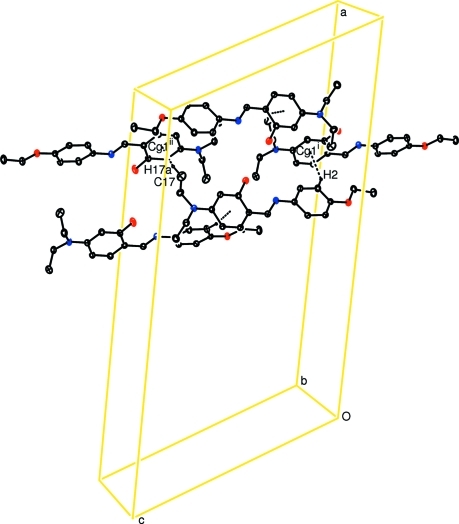
A packing diagram for (I). C—H···π interactions are drawn as dashed lines. [Symmetry codes: (i) x, 1 - y, -1/2 + z; (ii) 1/2 - x, 1/2 + y, 3/2 - z]

## supplementary crystallographic information

### Comment

Schiff bases are used as substrates in the preparation of number of industrial and biologically active compounds *via* ring closure, cycloaddition and replacement reactions. Some Schiff base derivatives are also known to have biological activities such as antimicrobial (El-Masry *et al.*, 2000; Pandeya *et al.*, 1999); antifungal (Singh & Dash 1988; Varma *et al.*, 1986) and antitumor (Hodnett & Dunn 1970; Misra *et al.*, 1981; Agarwal *et al.*, 1983). There are two characteristic properties of Schiff bases, *viz*. photochromism and thermochromism (Cohen *et al.*, 1964; Moustakali-Mavridis *et al.*, 1978). Schiff bases display two possible tautomeric form, namely the phenol-imine (O—H···N) and keto-amine (N—H···O) forms. In the solid state, the keto-amine tautomer has been found in naphthaldimines (Hökelek *et al.*, 2000; Odabaşoğlu *et al.*, 2003), while the phenol-imine form exists in salicylaldimine Schiff bases (Kaitner & Pavlovic, 1996; Yıldız *et al.*, 1998).

In the title compound, (I), the phenol-imine tautomer is favoured over the keto-amine form, and there is an intramolecular O—H···N hydrogen bond (Fig. 1 and Table 1). It is known that Schiff bases may exhibit thermochromism or photochromism, depending on the planarity or non-planarity of the molecule, respectively. This planarity of the molecule allows the H atom to be transferred through the hydrogen bond in the ground state with a low energy requirement (Hadjoudis *et al.*, 1987). Therefore, one can expect thermochromic properties in (I) caused by planarity of the molecule: the dihedral angle between rings A (C1—C6) and B (C8—C13) is 17.33 (16)° (Fig. 1). In (I), the C8—C7, C4—N1, C7=N1 and O1—C13 bond lengths of 1.441 (4), 1.417 (3), 1.263 (3) and 1.338 (3) Å, respectively are in good agreement with those observed in (*E*)-2[(3-Fluoropheng)iminomethy]-4-(trifluoromethoxy)phenol [1.447 (4), 1.420 (3), 1.268 (3) and 1.343 (3) Å, Bingöl Alpaslan *et al.*, 2010]. The C5—C4—N1=C7 and N1=C7—C8—C13 torsion angles are -19.0 (5)° and 1.2 (5)°, respectively. In crystal packing, the interactions [C2—H2···*Cg*1(*x*, 1 - *y*, *z* - 1/2)] and [C17—H17A···*Cg*1(1/2 - *x*, 1/2 + *y*, 3/2 - *z*)] are effective (Table 1 and Fig. 2.)

### Experimental

The title compound was prepared by refluxing a mixture of a solution containing 5-(diethylamino)-2-hydroxybenzaldehyde (0.5 g, 2.59 mmol) in 20 ml e thanol and a solution containing 4-ethoxyaniline (0.4 g, 2.59 mmol) in 20 ml e thanol. The reaction mixture was stirred for 1 h under reflux. The crystals of (*E*)-5-(diethylamino)-2-[(4-ethoxyphenylimino)methyl]phenol suitable for *x*-ray analysis were obtained by slow evaporation from ethyl alcohol (yield % 82;).

### Refinement

All H atoms were refined using a riding model with O—H=0.82 Å and C—H = 0.93 to 0.97 Å, and with *U*_iso_(H) = 1.2–1.5 *U*_eq_ (C,*O*).

### Crystal data

**Table:**

C_19_H_24_N_2_O_2_	*F*(000) = 1344
*M**_r_* = 312.40	*D*_x_ = 1.183 Mg m^−^^3^
Monoclinic, *C*2/*c*	Mo *K*α radiation, λ = 0.71073 Å
Hall symbol: -C 2yc	Cell parameters from 18643 reflections
*a* = 29.4936 (13) Å	θ = 1.5–28.0°
*b* = 7.8546 (2) Å	µ = 0.08 mm^−^^1^
*c* = 16.7146 (7) Å	*T* = 296 K
β = 115.093 (3)°	Prism, yellow
*V* = 3506.7 (2) Å^3^	0.76 × 0.59 × 0.28 mm
*Z* = 8	

### Data collection

**Table:**

Stoe IPDS 2 diffractometer	3625 independent reflections
Radiation source: fine-focus sealed tube	2383 reflections with *I* > 2σ(*I*)
graphite	*R*_int_ = 0.073
Detector resolution: 6.67 pixels mm^-1^	θ_max_ = 26.5°, θ_min_ = 1.5°
rotation method scans	*h* = −36→36
Absorption correction: integration (*X-RED32*; Stoe & Cie, 2002)	*k* = −9→9
*T*_min_ = 0.944, *T*_max_ = 0.979	*l* = −20→20
22701 measured reflections	

### Refinement

**Table:**

Refinement on *F*^2^	Primary atom site location: structure-invariant direct methods
Least-squares matrix: full	Secondary atom site location: difference Fourier map
*R*[*F*^2^ > 2σ(*F*^2^)] = 0.080	Hydrogen site location: inferred from neighbouring sites
*wR*(*F*^2^) = 0.260	H-atom parameters constrained
*S* = 1.10	*w* = 1/[σ^2^(*F*_o_^2^) + (0.1257*P*)^2^ + 1.7422*P*] where *P* = (*F*_o_^2^ + 2*F*_c_^2^)/3
3625 reflections	(Δ/σ)_max_ < 0.001
208 parameters	Δρ_max_ = 0.56 e Å^−^^3^
4 restraints	Δρ_min_ = −0.28 e Å^−^^3^

### Special details

**Table:**

Geometry. All e.s.d.'s (except the e.s.d. in the dihedral angle between two l.s. planes) are estimated using the full covariance matrix. The cell e.s.d.'s are taken into account individually in the estimation of e.s.d.'s in distances, angles and torsion angles; correlations between e.s.d.'s in cell parameters are only used when they are defined by crystal symmetry. An approximate (isotropic) treatment of cell e.s.d.'s is used for estimating e.s.d.'s involving l.s. planes.
Refinement. Refinement of *F*^2^ against ALL reflections. The weighted *R*-factor *wR* and goodness of fit *S* are based on *F*^2^, conventional *R*-factors *R* are based on *F*, with *F* set to zero for negative *F*^2^. The threshold expression of *F*^2^ > σ(*F*^2^) is used only for calculating *R*-factors(gt) *etc*. and is not relevant to the choice of reflections for refinement. *R*-factors based on *F*^2^ are statistically about twice as large as those based on *F*, and *R*- factors based on ALL data will be even larger.

### Fractional atomic coordinates and isotropic or equivalent isotropic displacement parameters (Å^2^)

**Table:**

	*x*	*y*	*z*	*U*_iso_*/*U*_eq_	
C1	0.55509 (10)	0.2708 (3)	0.09804 (16)	0.0622 (7)	
C2	0.59206 (13)	0.3870 (4)	0.14442 (19)	0.0790 (9)	
H2	0.6032	0.4636	0.1143	0.095*	
C3	0.61234 (13)	0.3892 (4)	0.23535 (19)	0.0788 (9)	
H3	0.6374	0.4677	0.2658	0.095*	
C4	0.59672 (10)	0.2789 (4)	0.28250 (17)	0.0633 (7)	
C5	0.55981 (11)	0.1594 (4)	0.23483 (18)	0.0700 (7)	
H5	0.5489	0.0815	0.2648	0.084*	
C6	0.53963 (10)	0.1569 (4)	0.14410 (17)	0.0688 (7)	
H6	0.5152	0.0770	0.1133	0.083*	
C7	0.60388 (11)	0.2229 (4)	0.42580 (18)	0.0677 (7)	
H7	0.5730	0.1684	0.3999	0.081*	
C8	0.62939 (10)	0.2270 (3)	0.52080 (17)	0.0634 (7)	
C9	0.60954 (11)	0.1486 (4)	0.57326 (18)	0.0734 (8)	
H9	0.5786	0.0950	0.5459	0.088*	
C10	0.63345 (11)	0.1467 (4)	0.66328 (18)	0.0706 (8)	
H10	0.6190	0.0904	0.6957	0.085*	
C11	0.68015 (11)	0.2300 (4)	0.70765 (17)	0.0663 (7)	
C12	0.69978 (11)	0.3123 (4)	0.65580 (18)	0.0737 (8)	
H12	0.7299	0.3706	0.6832	0.088*	
C13	0.67567 (11)	0.3099 (4)	0.56443 (17)	0.0668 (7)	
C14	0.68851 (12)	0.1161 (5)	0.85131 (19)	0.0863 (10)	
H14A	0.7178	0.0829	0.9037	0.104*	
H14B	0.6739	0.0137	0.8179	0.104*	
C15	0.65140 (15)	0.1970 (5)	0.8787 (3)	0.1017 (12)	
H15A	0.6427	0.1182	0.9139	0.153*	
H15B	0.6219	0.2272	0.8271	0.153*	
H15C	0.6658	0.2975	0.9127	0.153*	
C16	0.74589 (14)	0.3596 (6)	0.8467 (2)	0.1112 (14)	
H16A	0.7407	0.4619	0.8114	0.133*	
H16B	0.7453	0.3906	0.9024	0.133*	
C17	0.79396 (19)	0.2842 (7)	0.8626 (3)	0.1395 (18)	
H17A	0.8202	0.3645	0.8931	0.209*	
H17B	0.7944	0.2546	0.8073	0.209*	
H17C	0.7990	0.1836	0.8981	0.209*	
C18	0.54784 (14)	0.3722 (5)	−0.04086 (19)	0.0911 (10)	
H18A	0.5835	0.3629	−0.0240	0.109*	
H18B	0.5406	0.4877	−0.0293	0.109*	
C19	0.51921 (17)	0.3309 (6)	−0.1370 (2)	0.1181 (15)	
H19A	0.5286	0.4088	−0.1716	0.177*	
H19B	0.4840	0.3409	−0.1531	0.177*	
H19C	0.5267	0.2167	−0.1478	0.177*	
N1	0.62123 (9)	0.2895 (3)	0.37583 (14)	0.0701 (6)	
N2	0.70399 (10)	0.2291 (4)	0.79754 (15)	0.0912 (9)	
O1	0.69745 (9)	0.3886 (3)	0.51884 (14)	0.1011 (9)	
H1	0.6798	0.3787	0.4658	0.152*	
O2	0.53303 (8)	0.2546 (3)	0.00831 (12)	0.0780 (6)	

### Atomic displacement parameters (Å^2^)

**Table:**

	*U*^11^	*U*^22^	*U*^33^	*U*^12^	*U*^13^	*U*^23^
C1	0.0630 (15)	0.0717 (16)	0.0496 (13)	0.0059 (13)	0.0218 (11)	−0.0012 (11)
C2	0.100 (2)	0.0798 (19)	0.0593 (16)	−0.0170 (17)	0.0355 (16)	0.0001 (13)
C3	0.093 (2)	0.0834 (19)	0.0564 (15)	−0.0248 (17)	0.0281 (14)	−0.0084 (14)
C4	0.0642 (15)	0.0705 (16)	0.0532 (14)	0.0001 (13)	0.0230 (12)	−0.0035 (11)
C5	0.0692 (16)	0.0829 (18)	0.0597 (15)	−0.0066 (14)	0.0291 (13)	0.0031 (13)
C6	0.0581 (15)	0.0851 (19)	0.0577 (15)	−0.0057 (13)	0.0191 (12)	−0.0052 (13)
C7	0.0663 (16)	0.0733 (17)	0.0603 (15)	−0.0030 (13)	0.0237 (13)	−0.0049 (13)
C8	0.0670 (16)	0.0653 (15)	0.0553 (14)	0.0001 (12)	0.0232 (12)	−0.0032 (11)
C9	0.0678 (17)	0.088 (2)	0.0616 (16)	−0.0118 (15)	0.0251 (13)	−0.0030 (14)
C10	0.0711 (17)	0.0843 (19)	0.0556 (14)	−0.0087 (14)	0.0260 (13)	0.0004 (13)
C11	0.0744 (17)	0.0709 (16)	0.0517 (14)	−0.0023 (13)	0.0249 (13)	−0.0013 (12)
C12	0.0741 (18)	0.0820 (19)	0.0599 (16)	−0.0155 (15)	0.0234 (14)	−0.0037 (14)
C13	0.0798 (18)	0.0651 (15)	0.0578 (15)	−0.0106 (13)	0.0315 (14)	−0.0003 (12)
C14	0.085 (2)	0.109 (2)	0.0555 (15)	−0.0049 (18)	0.0209 (15)	0.0098 (16)
C15	0.124 (3)	0.105 (3)	0.089 (2)	−0.011 (2)	0.058 (2)	0.000 (2)
C16	0.088 (2)	0.166 (4)	0.0653 (19)	−0.036 (2)	0.0193 (17)	0.011 (2)
C17	0.133 (4)	0.130 (4)	0.129 (4)	0.008 (3)	0.030 (3)	0.023 (3)
C18	0.109 (3)	0.106 (2)	0.0574 (16)	−0.002 (2)	0.0342 (17)	0.0046 (16)
C19	0.136 (3)	0.154 (4)	0.0554 (18)	−0.009 (3)	0.031 (2)	0.001 (2)
N1	0.0841 (16)	0.0725 (14)	0.0520 (12)	−0.0050 (12)	0.0273 (12)	−0.0018 (10)
N2	0.0797 (16)	0.137 (2)	0.0484 (13)	−0.0196 (15)	0.0187 (11)	0.0094 (13)
O1	0.1140 (18)	0.1272 (19)	0.0608 (12)	−0.0550 (16)	0.0358 (12)	−0.0062 (12)
O2	0.0849 (14)	0.0925 (14)	0.0498 (10)	−0.0046 (11)	0.0220 (9)	0.0006 (9)

### Geometric parameters (Å, °)

**Table:**

C1—O2	1.364 (3)	C12—H12	0.9300
C1—C6	1.378 (4)	C13—O1	1.338 (3)
C1—C2	1.381 (4)	C14—N2	1.467 (4)
C2—C3	1.377 (4)	C14—C15	1.495 (5)
C2—H2	0.9300	C14—H14A	0.9700
C3—C4	1.375 (4)	C14—H14B	0.9700
C3—H3	0.9300	C15—H15A	0.9600
C4—C5	1.402 (4)	C15—H15B	0.9600
C4—N1	1.417 (3)	C15—H15C	0.9600
C5—C6	1.374 (4)	C16—C17	1.453 (5)
C5—H5	0.9300	C16—N2	1.547 (5)
C6—H6	0.9300	C16—H16A	0.9700
C7—N1	1.263 (4)	C16—H16B	0.9700
C7—C8	1.441 (4)	C17—H17A	0.9600
C7—H7	0.9300	C17—H17B	0.9600
C8—C9	1.388 (4)	C17—H17C	0.9600
C8—C13	1.405 (4)	C18—O2	1.423 (4)
C9—C10	1.364 (4)	C18—C19	1.499 (4)
C9—H9	0.9300	C18—H18A	0.9700
C10—C11	1.417 (4)	C18—H18B	0.9700
C10—H10	0.9300	C19—H19A	0.9600
C11—N2	1.362 (3)	C19—H19B	0.9600
C11—C12	1.389 (4)	C19—H19C	0.9600
C12—C13	1.385 (4)	O1—H1	0.8200
			
O2—C1—C6	115.9 (2)	C15—C14—H14A	109.0
O2—C1—C2	125.0 (3)	N2—C14—H14B	109.0
C6—C1—C2	119.0 (2)	C15—C14—H14B	109.0
C3—C2—C1	119.9 (3)	H14A—C14—H14B	107.8
C3—C2—H2	120.1	C14—C15—H15A	109.5
C1—C2—H2	120.1	C14—C15—H15B	109.5
C4—C3—C2	122.0 (3)	H15A—C15—H15B	109.5
C4—C3—H3	119.0	C14—C15—H15C	109.5
C2—C3—H3	119.0	H15A—C15—H15C	109.5
C3—C4—C5	117.7 (2)	H15B—C15—H15C	109.5
C3—C4—N1	117.1 (2)	C17—C16—N2	109.0 (4)
C5—C4—N1	125.2 (3)	C17—C16—H16A	109.9
C6—C5—C4	120.4 (3)	N2—C16—H16A	109.9
C6—C5—H5	119.8	C17—C16—H16B	109.9
C4—C5—H5	119.8	N2—C16—H16B	109.9
C5—C6—C1	121.1 (3)	H16A—C16—H16B	108.3
C5—C6—H6	119.5	C16—C17—H17A	109.5
C1—C6—H6	119.5	C16—C17—H17B	109.5
N1—C7—C8	123.4 (3)	H17A—C17—H17B	109.5
N1—C7—H7	118.3	C16—C17—H17C	109.5
C8—C7—H7	118.3	H17A—C17—H17C	109.5
C9—C8—C13	117.1 (2)	H17B—C17—H17C	109.5
C9—C8—C7	121.6 (3)	O2—C18—C19	107.9 (3)
C13—C8—C7	121.4 (3)	O2—C18—H18A	110.1
C10—C9—C8	122.8 (3)	C19—C18—H18A	110.1
C10—C9—H9	118.6	O2—C18—H18B	110.1
C8—C9—H9	118.6	C19—C18—H18B	110.1
C9—C10—C11	120.3 (3)	H18A—C18—H18B	108.4
C9—C10—H10	119.8	C18—C19—H19A	109.5
C11—C10—H10	119.8	C18—C19—H19B	109.5
N2—C11—C12	122.3 (3)	H19A—C19—H19B	109.5
N2—C11—C10	120.5 (3)	C18—C19—H19C	109.5
C12—C11—C10	117.3 (2)	H19A—C19—H19C	109.5
C13—C12—C11	121.8 (3)	H19B—C19—H19C	109.5
C13—C12—H12	119.1	C7—N1—C4	122.9 (3)
C11—C12—H12	119.1	C11—N2—C14	122.0 (3)
O1—C13—C12	118.4 (3)	C11—N2—C16	120.3 (3)
O1—C13—C8	120.9 (2)	C14—N2—C16	117.5 (2)
C12—C13—C8	120.7 (3)	C13—O1—H1	109.5
N2—C14—C15	112.9 (3)	C1—O2—C18	117.0 (2)
N2—C14—H14A	109.0		
			
O2—C1—C2—C3	−178.6 (3)	C11—C12—C13—C8	1.7 (5)
C6—C1—C2—C3	−1.0 (5)	C9—C8—C13—O1	179.9 (3)
C1—C2—C3—C4	−0.4 (5)	C7—C8—C13—O1	0.3 (4)
C2—C3—C4—C5	1.5 (5)	C9—C8—C13—C12	−0.2 (4)
C2—C3—C4—N1	178.2 (3)	C7—C8—C13—C12	−179.8 (3)
C3—C4—C5—C6	−1.3 (4)	C8—C7—N1—C4	177.4 (3)
N1—C4—C5—C6	−177.8 (3)	C3—C4—N1—C7	164.5 (3)
C4—C5—C6—C1	0.0 (5)	C5—C4—N1—C7	−19.0 (5)
O2—C1—C6—C5	179.0 (3)	C12—C11—N2—C14	−167.9 (3)
C2—C1—C6—C5	1.1 (4)	C10—C11—N2—C14	12.7 (5)
N1—C7—C8—C9	−178.4 (3)	C12—C11—N2—C16	17.2 (5)
N1—C7—C8—C13	1.2 (5)	C10—C11—N2—C16	−162.2 (3)
C13—C8—C9—C10	−1.4 (4)	C15—C14—N2—C11	−92.0 (4)
C7—C8—C9—C10	178.3 (3)	C15—C14—N2—C16	83.0 (4)
C8—C9—C10—C11	1.4 (5)	C17—C16—N2—C11	−93.3 (4)
C9—C10—C11—N2	179.6 (3)	C17—C16—N2—C14	91.6 (4)
C9—C10—C11—C12	0.2 (4)	C6—C1—O2—C18	179.2 (3)
N2—C11—C12—C13	178.9 (3)	C2—C1—O2—C18	−3.1 (4)
C10—C11—C12—C13	−1.7 (5)	C19—C18—O2—C1	179.9 (3)
C11—C12—C13—O1	−178.4 (3)		

### Hydrogen-bond geometry (Å, °)

**Table:**

Cg1 is the centroid of C8–C13 ring.

**Table:**

*D*—H···*A*	*D*—H	H···*A*	*D*···*A*	*D*—H···*A*
O1—H1···N1	0.82	1.88	2.610 (3)	148
C2—H2···Cg1^i^	0.93	2.85	3.681 (4)	149
C17—H17A···Cg1^ii^	0.96	2.97	3.763 (6)	140

Symmetry codes: (i) *x*, −*y*+1, *z*−1/2; (ii) −*x*+1/2, *y*+1/2, −*z*+3/2.
